# The Pathogen- and Incidence-Based DALY Approach: An Appropriated Methodology for Estimating the Burden of Infectious Diseases

**DOI:** 10.1371/journal.pone.0079740

**Published:** 2013-11-20

**Authors:** Marie-Josée J. Mangen, Dietrich Plass, Arie H. Havelaar, Cheryl L. Gibbons, Alessandro Cassini, Nikolai Mühlberger, Alies van Lier, Juanita A. Haagsma, R. John Brooke, Taavi Lai, Chiara de Waure, Piotr Kramarz, Mirjam E. E. Kretzschmar, on behalf of the BCoDE consortium

**Affiliations:** 1 Julius Centre for Health Sciences and Primary Care, University Medical Centre Utrecht, Utrecht, The Netherlands; 2 Department of Public Health Medicine, School of Public Health, University of Bielefeld, Bielefeld, Germany; 3 Centre for Infectious Disease Control, National Institute for Public Health and the Environment, Bilthoven, The Netherlands; 4 Institute for Risk Assessment Sciences, Utrecht University, Utrecht, The Netherlands; 5 Centre for Immunity, Infection and Evolution, Institute for Immunology and Infection Research, School of Biological Sciences, University of Edinburgh, Edinburgh, United Kingdom; 6 European Centre for Disease Prevention and Control, Stockholm, Sweden; 7 Institute of Public Health, Medical Decision Making and Health Technology Assessment, Department of Public Health and Health Technology Assessment, UMIT - University for Health Sciences, Medical Informatics and Technology, Hall in Tirol, Austria; 8 Public Health, Erasmus University Medical Center, Rotterdam, The Netherlands; 9 Department of Public Health, University of Tartu, Tartu, Estonia; 10 Institute of Public Health, Catholic University of the Sacred Heart, Rome, Italy; The Australian National University, Australia

## Abstract

In 2009, the European Centre for Disease Prevention and Control initiated the ‘Burden of Communicable Diseases in Europe (BCoDE)’ project to generate evidence-based and comparable burden-of-disease estimates of infectious diseases in Europe. The burden-of-disease metric used was the Disability-Adjusted Life Year (DALY), composed of years of life lost due to premature death (YLL) and due to disability (YLD). To better represent infectious diseases, a pathogen-based approach was used linking incident cases to sequelae through outcome trees. Health outcomes were included if an evidence-based causal relationship between infection and outcome was established. Life expectancy and disability weights were taken from the Global Burden of Disease Study and alternative studies. Disease progression parameters were based on literature. Country-specific incidence was based on surveillance data corrected for underestimation. Non-typhoidal *Salmonella* spp. and *Campylobacter* spp. were used for illustration. Using the incidence- and pathogen-based DALY approach the total burden for *Salmonella* spp. and *Campylobacter* spp. was estimated at 730 DALYs and at 1,780 DALYs per year in the Netherlands (average of 2005–2007). Sequelae accounted for 56% and 82% of the total burden of *Salmonella* spp. and *Campylobacter* spp., respectively. The incidence- and pathogen-based DALY methodology allows in the case of infectious diseases a more comprehensive calculation of the disease burden as subsequent sequelae are fully taken into account. Not considering subsequent sequelae would strongly underestimate the burden of infectious diseases. Estimates can be used to support prioritisation and comparison of infectious diseases and other health conditions, both within a country and between countries.

## Introduction

The disability-adjusted life year (DALY), a metric quantifying and combining the impact of premature death and non-fatal health outcomes resulting from disease, was jointly developed by the World Bank, Harvard School of Public Health and the World Health Organization for the Global Burden of Disease and injury (GBD) study [1]–[4]. DALYs were developed with the aim of supporting priority setting for healthcare and health research, to identify disadvantaged groups for targeted healthcare interventions, and to provide a comparable output measure for interventions, evaluations and planning [4]. Since their development and introduction in the World Development Report [5] DALYs have been widely used in both national and global disease burden estimations (e.g. [6]–[10]).

In Europe, infectious diseases were estimated to account for less than 10% of the total burden of disease as measured by DALYs [11]. However, this figure might underestimate the true burden of infectious diseases as subsequent sequelae were not fully taken into account in most previously conducted disease burden assessments [12]. To get a better insight into the true disease burden in Europe [13]–[15], the European Centre for Disease Prevention and Control (ECDC) launched the Burden of Communicable Diseases in the European Union, EEA and EFTA countries (BCoDE) project in 2009. This project aims to generate evidence-based, robust and comparable disease burden estimates of infectious diseases in Europe. The methodology applied is presented in this current paper, using non-typhoidal *Salmonella* spp. and *Campylobacter* spp. for illustration.

## Methodology

The term “Burden of disease” refers to a quantitative estimation of the impact of diseases on a population or geographical region, using a multitude of indicators. For the BCoDE-project, a disease burden-indicator was needed that captures and weighs the impact of acute illness due to infectious diseases and associated sequelae on morbidity and mortality in a single metric, thereby allowing the comparison between infectious diseases within and between countries, and with other health conditions. In addition, methods adopted by the BCoDE-project should allow future development towards a methodology that accounts for the dynamic nature of infectious diseases and impact of intervention(s) [12]. As economic analyses were not foreseen, non-monetary measures were favoured; of these Quality-Adjusted Life Years (QALY) and Disability-Adjusted Life Years (DALY) are the most prominent metrics [16]–[19]. QALYs are used in health economics in high-income countries (including Europe) [20]. DALYs are used worldwide, for all age-classes and various health conditions [21]. QALYs represent survival that is down-weighed for the time lived with functional capacity, whereas DALYs are a health gap measure which directly quantifies health loss, and therefore is a more straightforward burden of disease measure, underpinning our choice for the DALY methodology.

### Disability-Adjusted Life Years

The DALY was introduced by Murray and co-workers in the GBD-study [4], [22]. It is composed of a measure for the number of years of life lost due to premature death (YLL) and the number of years of life lost due to disability (YLD). The DALY, as a normative measure, quantifies the health losses (in years) based on the difference between the observed and ideally expected population-based health goal [3]. YLD are computed by weighing each remaining life year with a factor (disability weight) between 0 (perfect health) and 1 (death) depending on the severity of the concerning disability.

YLD are calculated as the product of the duration of the illness (*t*) and the disability weights (*w*) of a specific health outcome, accumulated over the number of incident cases (*n*) of all health outcomes (*l*):
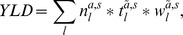
where *t*, *w* and *n* for health outcome *l* may be age-dependent (*a*) and/or sex-dependent (*s*), where *a* stands for age at infection and *ã* for age at disease onset and death. For more details see File S1.

YLL for a specific health outcome are calculated by summation of the number of all fatal cases (*d*) due to the health outcome (*l*) at age (*a*), each case multiplied by the remaining individual life expectancy (*e*) at the age of death *ã*. *d* for health outcome *l* may be age-dependent (*a*) and-or sex-dependent (*s*). *e* is by definition age- and sex-dependent. Thus:
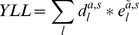



The DALY is then calculated as the sum of the YLL and YLD (for more details File S1).

Applying the DALY methodology requires decisions reflecting on value choices. To assure comparability to other disease burden-assessments all YLL estimates are based on the Coale and Demeny West Level 26 and 25 life tables. Regarding disability weights, we relied on the GBD-study, where available, supplemented by disability weights from other studies with methods similar to the GBD assessments [23]. Durations of health outcomes, possibly age- and/or sex-dependent, were based on published literature. Time-discounting and age-discounting was not applied.

### Incidence- and Pathogen-based DALY

The DALY can be calculated using different methodological approaches. The correct assignment of disease burden to the causal event is important for the estimation of disease burden for infectious diseases in order to provide thorough and reliable estimates. To attribute all health consequences of an infection to the initial infectious event, and therefore estimate the complete burden caused by this infection decisions are required on whether or not sequelae are causally linked to the infection. In the incidence- and pathogen-based DALY approach [12]–[14], [24]–[26] sequelae, for which there is sufficient evidence for causal relationship, are related to the initial infection by means of an outcome tree representing the natural history of the infection and its short- and long-term sequelae (File S1).

### Outcome Tree

**Figure 1:**
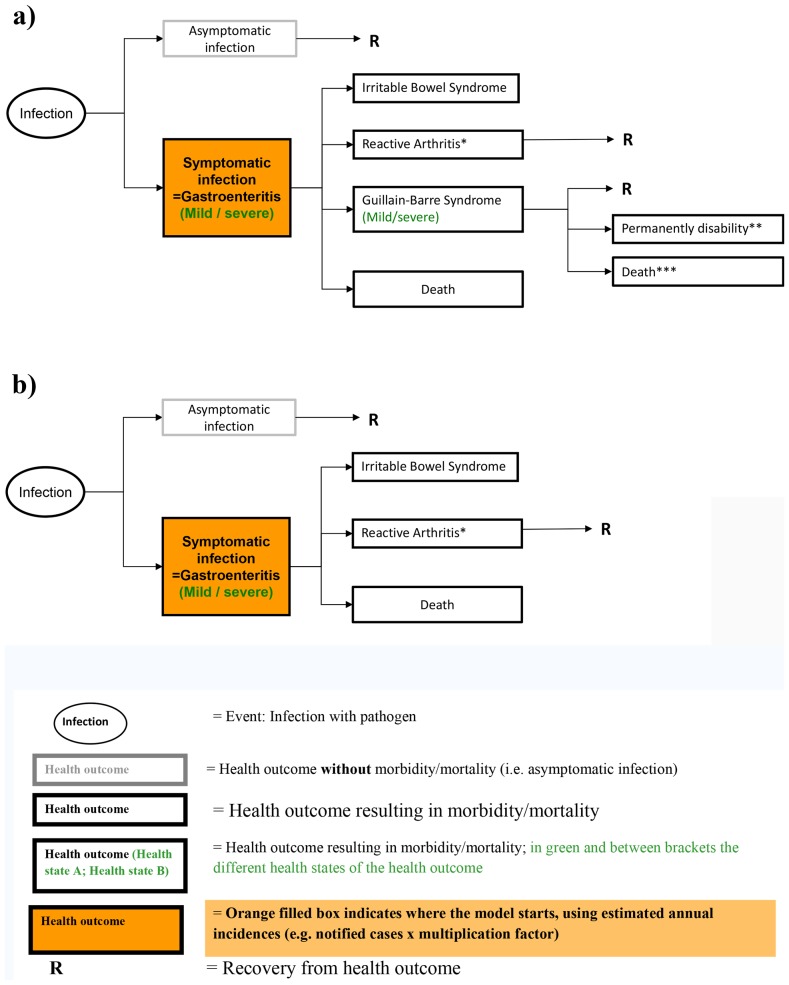
Outcome tree for *Campylobacter* spp. (a) and *Salmonella* spp. (b) – an illustration. Note: *Only severe GE cases are at risk to develop ReA. ** Non-fatal severe GBS cases may develop permanent disability. *** For reasons of simplicity we assume that only severe GBS cases may be fatal.

In order to assess the disease burden for the selected pathogens, - in total 32 pathogens (see Kretzschmar *et al.* [12]) -, the different health outcomes following infection with a particular agent were defined. These health outcomes were described in the format of an outcome tree (Figure 1) which provides a qualitative representation of the disease progression pathways over time by ordering all relevant health outcomes following infection and illustrating their conditional dependencies (File S1). Using the incidence- and pathogen-based DALY approach, all DALYs associated with current and future health outcomes are assigned to the year of the initial infection (i.e. the first node of the tree).

In some instances, it was necessary to split a health outcome into subcategories or ‘health states’, according to their severity to better represent the true disease burden. We hereby distinguish between vertical and horizontal disaggregation (for more details see [23]). Vertical disaggregation is the distribution of *health states* related to a specific health outcome, all occurring at the same time period after infection, into subcategories describing the severity of the health outcome (e.g. mild and severe gastroenteritis (GE) (Figure 1)). Horizontal disaggregation describes different health outcomes occurring sequentially in time in one and the same person, which are clinically different conditions or diseases (e.g. Reactive arthritis (Figure 1)).

Choices made in the construction of the outcome trees for the 32 pathogens were based on systematic literature review. Sufficient evidence of a causal relationship or sufficient evidence of an association between an infection with a pathogen and sequela was required for inclusion into an outcome tree.

### Disease Burden-model

Incident cases of symptomatic infections at the root of the tree served as an input for our model, mostly extracted from (mandatory) surveillance data for notifiable infectious diseases or equivalent data sources. Input data has to be stratified by age and sex (File S1).

However, the incidence is likely not fully represented by raw data on notified cases owing to the probability of underestimation of infectious diseases. It is therefore crucial to identify areas and causes of underestimation and correct for underestimation to better estimate the incidence.

The overall extent of underestimation can be explained by two major effects represented by under-ascertainment and under-reporting [27]. Under-ascertainment refers to cases that do not seek healthcare advice [27]. Under-reporting, - also including under-diagnosis -, refers to cases that seek healthcare but for whom either a specimen was not collected, or for whom a specimen was collected but did not result in laboratory examination, or whose infection status was not (correctly) reported to national surveillance systems [27]–[28]. Within the BCoDE-project, corrections for underestimation are applied by using multiplication factor(s) (MF), representing either correction for underestimation in one step, or separate correction for under-ascertainment and under-reporting in two steps (see File S1).

The method used to estimate such MFs depends on the specific pathogens, the type of data available and the reasons for under-reporting and under-ascertainment. In general, MFs are developed by comparing incidence in the general population (determined by community-based or serological studies (for more details see [27])) with the number of notified cases extracted from national or supra-national databases. The choice of MFs was guided by information from published studies, and complemented by expert knowledge. MFs must be disease-specific (since under-reporting and under-ascertainment affects different diseases with varying magnitudes), country-specific (owing to variations in disease exposure, surveillance, laboratory practices and healthcare systems, availability of treatment, as well as cultural, social and technological differences), and age-specific (since rates of under-reporting and under-ascertainment can vary widely between age groups for many diseases), possibly sex-specific, and in some instances even strain-specific [27].

Having estimated the number of incident cases of symptomatic infections at the root of the tree, using raw input data adjusted by MFs (see File S1 for details), incident cases of the subsequent health outcomes were estimated throughout the outcome tree using the (conditional) probabilities of progressing from one stage to the next or to recovery (File S1). If asymptomatic cases also contribute to disease burden, than the number of incident cases of symptomatic infections was corrected by a factor *τ* to estimate the number of all infected cases. *τ* may be age- and/or sex-dependent (see File S1). Incident cases of the subsequent health outcomes resulting from asymptomatic cases were also estimated throughout the outcome tree using the (conditional) probabilities of progressing from one stage to the next or to recovery (File S1). Conditional probabilities were based on literature review, and may be age- and sex-dependent. Data necessary for quantitative estimates of disease burden estimates are often limited, fragmented or based on small samples, resulting in a considerable degree of uncertainty. Parameters representing such a lack of perfect knowledge (i.e. MFs and (conditional) transition probabilities) were explicitly modelled by incorporating probability distributions, using either uniform or pert distributions (note Pert distribution is a specific beta distribution used in risk analysis [29]), and the Monte Carlo simulation technique to estimate predictive intervals. However, for parameters that represent inherent heterogeneity of a system as e.g. differences among patients (i.e. disability weights and duration of health outcomes), it was decided to use point estimates (i.e. average). Systematic uncertainty and uncertainty due to lack of data were explored by sensitivity analysis.

The outcomes of the models are disease burden estimates expressed in DALYs per year associated with infection with particular infectious agents and its related sequelae in a particular country. Data are presented both in aggregated form (DALYs per year) and in disaggregated form (YLD per year and YLL per year), from a population perspective (DALY/YLL/YLD per year per country and per 100,000 population per country) and from an individual perspective (DALY/YLL/YLD per year per each infected case). Using a population perspective, DALYs are stratified into age-classes and sex-classes, and into acute illness and sequelae.

### Data and Data Availability

Mandatory surveillance data for notifiable infectious diseases or equivalent data sources extracted from national or supra-national databases (including ECDC databases) and stratified by age and sex, served as input for our model (File S1). But other data sources (e.g. own incident estimates (for example using an attack rate to estimate symptomatic influenza cases); hospitalization data; mortality data (e.g. variant Creutzfeldt-Jakob disease)) may also be used as model input in these models. Correction for underestimation was nearly always required for the selected pathogens.

**Figure 2:**
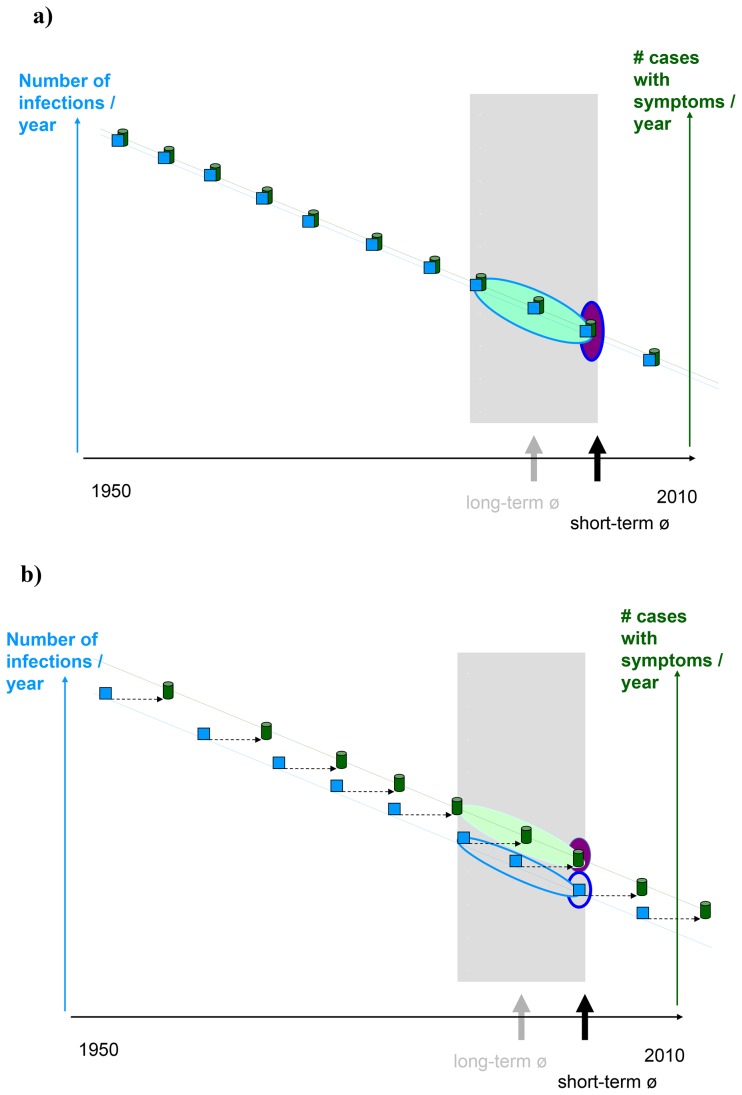
Assuming a downwards time trend for an infection having symptoms in the same years (a) and for an infection where symptoms occur only after 10 years (b). Note: Blue rectangles represent the number of infections in the year of infection (exposure to an infection). Green “cans” represent the number of cases with symptoms; where these symptomatic cases occur in the same year as the infection (a.) or a few years later (b.) as indicated by the dashed arrow. The long-term average (e.g. 10-year average) is highlighted by a light blue oval for incidence, and by a light green oval for prevalence. The short-term average (e.g. 3-year average) is represented by a dark blue oval for incidence and purple oval for prevalence.

Infectious diseases differ in their long-term dynamics and may display distinct time trends in incidence over years. Incidence of infectious diseases rarely remains constant for long-time periods, but decreases (increases) or oscillates over the years, or may have temporal peaks during outbreak situations. If there is a monotonically decreasing or increasing time trend in incidence, long-term averages would overestimate or underestimate the disease burden (see Figure 2.A). And for an infectious agent with an incubation period and/or latent phase longer than one year (in Figure 2.B for illustration ∼ 10 years) prevalence data rather than incidence data from acute infections would lead to an additional overestimation or underestimation. Therefore to minimise influence of long-term trends on estimates we based incidence estimates on data from a 3-year time period. However, for infections with irregular (non-monotonic) time trends over one or several years, or for diseases that occur in incidental outbreaks a long-term average (i.e. 10 years) was considered more representative for expected annual cases.

### Application of the Methods for Estimating the Disease Burden for Campylobacter spp. and Non-typhoidal Salmonella spp. in the Netherlands

Estimation of the disease burden associated with *Campylobacter* spp. and non-typhoidal *Salmonella spp.* in the Netherlands for the average of the years 2005–2007 illustrates the presented methodology.

Both symptomatic Campylobacter and Salmonella infections in humans most often result in acute but self-limiting gastroenteritis (GE) which resolves within a few days, but occasionally GE can be fatal. Reactive arthritis (ReA), Irritable Bowel Syndrome (IBS) and Guillain-Barré Syndrome (GBS) are the most frequently observed sequelae of *Campylobacter* spp. [25]–[26], [30] and were included in our outcome tree (Figure 1). ReA and IBS are the observed sequelae of non-typhoidal *Salmonella* spp. [30]–[33] (Figure 1). The probability of developing other post-infectious complications is low and was therefore disregarded. Given that the duration of diarrhoea correlates with the risk of developing ReA [34] and that most evidence on ReA is collected from GE cases requiring medical service we assume only severe GE cases are at risk of developing ReA [35]. But given the uncertainty of who is at risk of developing ReA, sensitivity analysis was conducted, assuming that all GE cases would be at risk of developing ReA.

Asymptomatic infections with Campylobacter and Salmonella do not lead to acute illness or sequelae, and are therefore not considered in our outcome trees. The conditional probabilities for different health outcomes, following the progression through the outcome tree, and the distribution of health states over health outcomes as used in the current study, are summarized in File S2.

Incident symptomatic *Campylobacter* spp. cases and incident symptomatic non-typhoidal *Salmonella* spp. cases were estimated, using data from a Dutch sentinel laboratory-surveillance system [36] (Wilfrid van Pelt, pers. communication; November 2011) and adjusted for underestimation with country- and pathogen-specific MFs based on a Swedish travellers study [37]. In this study, Havelaar *et al*. [37] calculated incidence rates of Campylobacter and Salmonella infections and MFs for all European countries based on disease risks of returning Swedish travellers, anchored to the Dutch population-based study on gastroenteritis; the Sensor-study. The MF used for *Campylobacter* spp. incident cases was sampled from a pert-distribution with most likely 18.85; minimum 7.4 and maximum 47.4 [37]. And the used MF for non-typhoidal *Salmonella* spp. incident cases was sampled from a pert-distribution with most likely 19.8; minimum 4.4 and maximum 64.8. These MFs corrected for underestimation (under-ascertainment and under-reporting) and for coverage of the sentinel surveillance system, which was 52% (*Campylobacter* spp.) and 64% (non-typhoidal *Salmonella* spp.) [37].

Incident cases of other health outcomes were estimated following the outcome tree using conditional probabilities (File S2). The disease burden-model was implemented in Microsoft Excel using @Risk and was run with 10,000 iterations. The results present the mean and the associated uncertainty boundaries of the 2.5^th^- and 97.5^th^-percentiles of the posterior distributions of outcome variables.

## Results

**Figure 3:**
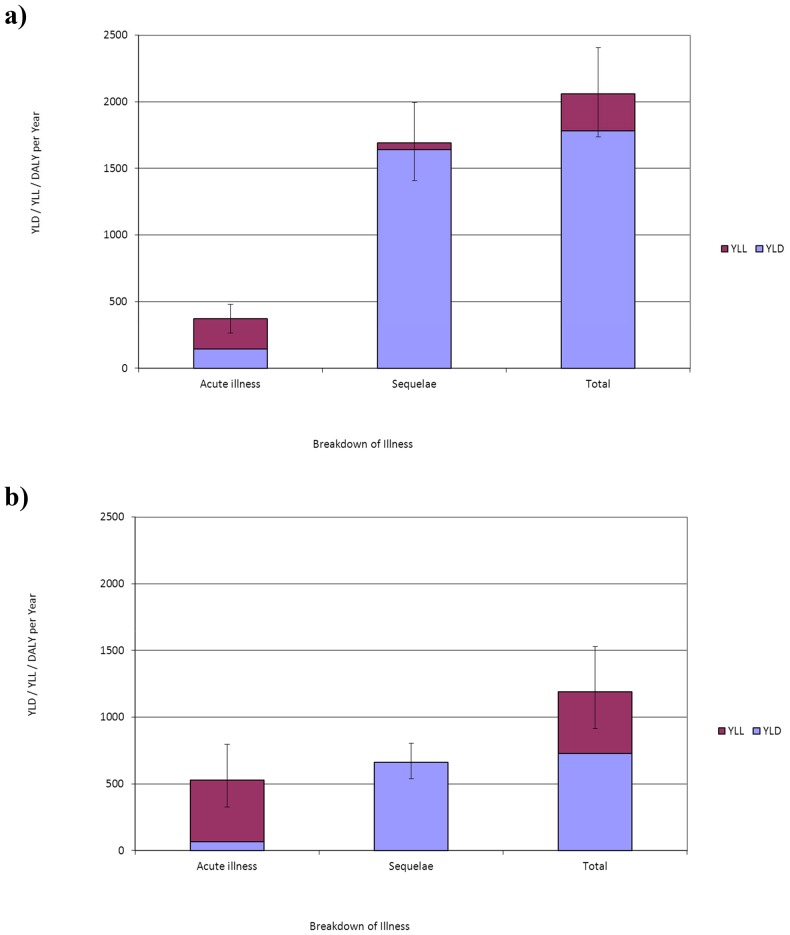
The undiscounted average burden of *Campylobacter* spp. (a) and *Salmonella* spp. (b) in the Netherlands (average of 2005–2007) in DALY per year, subdivided in YLL and YLD for acute illness, sequelae and total. The 95% uncertainty range is shown using error bars.

The estimated numbers of incident cases of *Campylobacter* spp. and non-typhoidal *Salmonella* spp. were on average 76,520 (95%C.I.: 67,790–85,550) and 35,300 (95%C.I.: 29,250–41,680) per year in the Netherlands for 2005–2007. Besides acute illness, both pathogens caused associated numbers of sequelae, on average 6,730 IBS cases (95%C.I.:5,780–7,790), 580 ReA cases (95%C.I.: 335–890) and 42 GBS cases (95%C.I.: 18–67) for *Campylobacter* spp. and 3,100 IBS cases (95%C.I.:2,520–3,760) and 183 ReA cases (95%C.I.: 110–270) for non-typhoidal *Salmonella* spp., respectively. The total disease burden for *Campylobacter* spp. and non-typhoidal *Salmonella* spp., respectively was on average 2,060 DALYs per year (95%C.I.: 1,740–2,410) and 1,190 DALYs per year (95%C.I.: 910–1,530). Sequelae-associated burden of disease accounted for 82% and 56% of the total disease burden for *Campylobacter* spp. and non-typhoidal *Salmonella* spp. (Figure 3). Results are presented in File S2.

**Figure 4:**
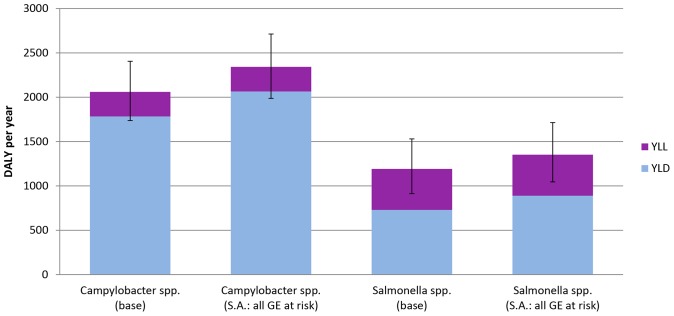
The undiscounted average burden of *Campylobacter* spp. and *Salmonella* spp. in the Netherlands (average of 2005–2007) in DALY per year, for base case and scenario analysis. DALY are subdivided in YLL and YLD for actue illness, sequelae and total. The 95% uncertainty range is shown using error bars. **Note:** “Base case” represents a situation where only severe GE cases are at risk to develop reactive arthritis (ReA). “SA: ReA” represents the scenario analysis where all GE cases are at risk to develop ReA.

If we assume that all GE cases rather than only severe GE cases are at risk to develop ReA the disease burden increases by 114% for *Campylobacter* spp. and by 113% for non-typhoidal *Salmonella* spp., respectively (Figure 4).

## Discussion

### The Methodology

As shown here, the proposed incident- and pathogen-based approach allows for a comprehensive estimation of the total disease burden caused by infection with a pathogen. For some infectious diseases the burden associated with sequelae is higher than that caused by acute illness. Therefore not considering these sequelae can therefore lead to considerable underestimation of the total disease burden, as was the case in most previously conducted disease burden assessments. Furthermore, the methodology presented here enables comparisons of disease burden estimates between infectious diseases, between countries, and with other health conditions. In this way the estimates can be used to identify key drivers of infectious disease burden and for priority settings.

The new GBD 2010 study presents prevalence-based DALY and the authors argue that this approach is more appropriate, because a) if incidence has declined substantially but prevalence is still high, this might lead to disadvantages in priority setting for those diseases, b) the incidence of some chronic conditions is not precisely measurable (e.g. the start of ischemic heart disease), and c) the incorporation of co-morbidity is more straightforward [38]. For health services use the prevalence-based DALYs might have some advantages above our approach. Both methods, if then accurately modeled, reflect disease burden at a particular point in time, but from different perspective. However, in case of infectious pathogens, and in particular for priority settings of interventions to prevent primary infections, we believe that incidence is the more suitable input for the DALY metric, because only with the initial start of the infection is it possible to include all disease sequelae that result from infection. In particular for infectious diseases with an upwards or downwards time trend and with an incubation period and/or latent phase longer than one year, using prevalence data from sequelae rather than incidence data from acute infections lead to additional overestimation or and underestimation of burden estimation as was demonstrated in Figure 2. The incidence- and pathogen based-outcome tree approach avoids the issue of attributing a proportion of the prevalence of sequelae with multiple potential causes, such as GBS. The incidence and pathogen-based approach furthermore allows a proper prediction of potential effects of interventions aiming at preventing infections, which is the primary focus in decision making. Using the prevalence-approach here would lead to different infection times of infected individuals within the outcome tree making proper predictions of intervention effects impossible. A last but important issue is that most surveillance systems for infectious diseases report incidence and being aware of the possibility to derive prevalence from incidence, it was decided to use incidence as the appropriate input.

We are not the first using an incidence-based approach (e.g. [39]–[42]). In case of infectious diseases most studies, however derived the incident cases of subsequent health outcomes using syndrome surveillance combined with etiological fractions, rather than using conditional probabilities (e.g. GBS [30], [43]), or if using conditional probabilities, not as extensively defined to include all relevant health outcomes by using conditional probabilities (e.g. [30], [43]). Deriving incident cases of subsequent health outcomes from syndrome surveillance is definitively a valid approach, but very time-consuming hampering annual updates of disease burden. In the approach presented in this paper, the explicit input in our disease burden model are the numbers of symptomatic infections. All subsequent health outcomes are modelled using conditional probabilities. This approach has as one major advantage, - presuming that conditional probabilities are properly defined initially -, namely that disease burden estimations of infectious diseases can easily be updated from year to year, and could therefore be used, - next to the annual reporting of surveillance data -, as additional information to decision makers. And with 32 pathogens in the toolkit that is currently being developed, health decision makers have evidence-based estimates to support prioritisation and comparison of infectious diseases.

This methodology also presents opportunities to possibly improve surveillance. In our experience, the collaboration between modellers and surveillance staff required to prepare burden estimates also identifies strengths and weakness of the existing surveillance system, prompting additional research.

### Estimated Disease Burden in the Netherlands

In the Netherlands sequelae-associated burden of disease contributed to more than 60% of the total disease burden for both *Campylobacter* spp. and non-typhoidal *Salmonella* spp. The incidence- and pathogen-based DALY approach presented here allows estimation of the complete burden caused by an infection and the attribution to specific short- and long-term sequelae.

Due to substantial uncertainties surrounding the MFs the uncertainty around the estimated incidence and consequently around the attributable disease burden is large for both pathogens.

The estimates are in line with Havelaar *et al.* [30], who obtained an average disease burden of 1,270 DALYs for non-typhoidal *Salmonella* spp. for 2009, using a comparable methodology. The disease burden estimates for *Campylobacter* spp. are slightly lower than found by Havelaar *et al.* [30] (3,250 DALYs), mainly due to the lower number of estimated incident cases in the years 2005–2007 as compared to 2009 (i.e. 90,000 cases).

With 2,060 DALYs (*Campylobacter* spp.) and 1,190 DALYs (non-typhoidal *Salmonella* spp.) per year, the impact on the total burden for Dutch society is relatively low compared to the burden of e.g. lung cancer (158,100 DALYs per year) and injuries (208,900 DALYs per year) [44]. Both are more comparable to AIDS with 3,800 DALYs per year and influenza with 8,600 DALYs per year [44].

### Potential Limitations of the Applied Methodology

Our approach has several potential limitations. Although estimates of MFs should be disease, country, age- and sex-specific relevant data is mostly missing or inconsistent, resulting in MF estimates that are often only disease- and country-specific. Consequently, the same MF is used for all age- and sex-classes. A potential drawback of using notified data, or their equivalents, corrected with MFs for estimating numbers of symptomatic incident cases is that those age- and sex-classes with relatively more notified severe cases are over-represented, and those age- and sex-classes with relatively fewer notified severe cases are under-represented. For health outcomes with short-term and self-limiting illnesses the numbers of incident cases are of major importance, and over-representation (under-representation) within specific age- and sex-classes is negligible. However, for infections with long-term sequelae an incorrect stratification of estimated incident cases over age- and sex-classes has an impact on the total disease burden estimates. Over-representing older age-classes, and under-representing younger age-classes might result in an underestimation of the estimated disease burden. Whereas under-representing older age-classes, and over-representing younger age-classes results in an overestimation of the total disease burden.

Estimates for MFs are ideally based on community-cohort studies, but even then uncertainties around the MF estimates are often huge, resulting in large uncertainty around the disease burden estimates.

Future research might lead to new evidence on the association of pathogens with other health outcome(s), and who is at risk of developing them. More research is necessary for some of the (conditional) transition probabilities which often remain highly uncertain.

## Conclusion

The methodological framework presented here is an important tool for generating comprehensive estimates of the disease burden of infectious diseases in Europe. It enables attribution of burden to short- and long-term sequelae and provides the basis for international comparison and prioritization of healthcare resources.

## Supporting Information

File S1 Figure S1– Outcome tree for an infectious pathogen – an illustration.
(PDF)

File S2 Figures Figure S2.1 Outcome tree for *Salmonella* spp. Figure S2.2 Outcome tree for *Campylobacter* spp. Figure S2.3 Assumed age-distribution of fatal GE cases (in percentage). Figure S2.4 Assumed age-distribution of GBS cases (in percentage), as derived from Havelaar et al. Figure S2.5 The average disease burden (DALY) of *Salmonella* spp. and associated sequelae in the Netherlands per age-group and gender. The 95% uncertainty range is shown using error bars. Figure S2.6 The average disease burden (DALY) of *Campylobacter* spp. in the Netherlands per age-group and gender. The 95% uncertainty range is shown using error bars. Figure S2.7 - The distribution of undiscounted average burden of Salmonella-associated sequeleae and Campylobacter-associated sequeale, respectively, over the associated sequelae. Tables Table S2.1 - Reported laboratory-confirmed cases for *Campylobacter* spp. and *Salmonella* spp. in the Netherlands (average of the years 2005–2007). Table S2.2 -Pathogen-specific multiplication factors for the Netherlands. Table S2.3 Percentages used in the *Salmonella* spp. outcome tree Table S2.4 Percentages used in the *Campylobacter* spp. outcome tree. Table S2.5 Disability weights and duration. Table S2.6: The disease burden of *Salmonella* spp and associated sequelae in the Netherlands – Summary results, average and 95% CI in brackets and italic. Table S2.7: The disease burden of *Campylobacter* spp. and associated sequelae in the Netherlands – Summary results, average and 95% CI in brackets and italic.
(PDF)
