# Correction: The Pathogen- and Incidence-Based DALY Approach: An Appropriated Methodology for Estimating the Burden of Infectious Diseases

**DOI:** 10.1371/annotation/caf33818-3453-4e30-b307-7526427b09b7

**Published:** 2013-12-20

**Authors:** Marie-Josée J. Mangen, Dietrich Plass, Arie H. Havelaar, Cheryl L. Gibbons, Alessandro Cassini, Nikolai Mühlberger, Alies van Lier, Juanita A. Haagsma, R. John Brooke, Taavi Lai, Chiara de Waure, Piotr Kramarz, Mirjam E. E. Kretzschmar, on behalf of the BCoDE consortium

The word "appropriate" is misspelled in the article title. The correct title is: The Pathogen- and Incidence-Based DALY Approach: An Appropriate Methodology for Estimating the Burden of Infectious Diseases.

The correct citation is: Mangen M-JJ, Plass D, Havelaar AH, Gibbons CL, Cassini A, et al. (2013) The Pathogen- and Incidence-Based DALY Approach: An Appropriate Methodology for Estimating the Burden of Infectious Diseases. PLoS ONE 8(11): e79740. doi:10.1371/journal.pone.0079740

In the Affiliation, the line "Membership of the Mouse Genome Sequencing Consortium is provided in the Acknowledgments" should read "Other members of the BCoDE consortium are listed in the Acknowledgments." 

