# Supplementary material for: The Pathogen- and Incidence-Based DALY Approach: An Appropriated Methodology for Estimating the Burden of Infectious Diseases
Source: PLoS One. 2013 Nov 20;8(11):e79740. doi: 10.1371/journal.pone.0079740 (PMC3835936; doi:10.1371/journal.pone.0079740)
Supplement: File S1 — Figure S1– Outcome tree for an infectious pathogen – an illustration. (PDF) [file pone.0079740.s001.pdf]

Supporting Information file 1. – Modelling the disease burden in DALYs using an incidence- and pathogen-based approach.

Having defined an outcome tree for a particular infectious pathogen (see figure S1), crude annual numbers of incident symptomatic cases (n_c) for the root health outcome l_r, stratified by sex s (s=0,1) and age at infection a (a=0-a_{max}), is extracted from surveillance data, and used as initial model input. Where necessary n_c has to be corrected for underestimation by a factor σ, estimated either directly, or as the product of a factor α (to correct for under-ascertainment) and a factor β (to correct for under-reporting). All three factors may be age-dependent and/or sex-dependent, and are pathogen- and country-specific. The number of incident symptomatic cases (n_e) for root health outcome l_r can then be estimated as:

$$n_e^{a,s} = n_c^{a,s} * \sigma^{a,s} = n_c^{a,s} * \left(\alpha^{a,s} * \beta^{a,s}\right).$$

Here n_e represents the number of symptomatically infected cases, but not all infected cases, as some infections may be asymptomatic. Therefore, if asymptomatic cases contribute to disease burden, n_e has to be corrected by a factor τ to estimate the number of all infected cases (n_t). τ may be age- and/or sex-dependent. Then the estimated number of all infected cases is:

$$n_t^{a,s} = n_e^{a,s} * \tau^{a,s} = \left(n_c^{a,s} * \sigma^{a,s}\right) * \tau^{a,s} = \left(n_c^{a,s} * \left(\alpha^{a,s} * \beta^{a,s}\right)\right) * \tau^{a,s}.$$

The number of asymptomatically infected incident cases (n_f) is then:

$$n_f^{a,s} = n_t^{a,s} - n_e^{a,s}.$$

We estimate the numbers of incident cases of sequelae and deaths using conditional probabilities of moving from one health outcome (l) to another, taking into account the sequence of events as illustrated in the outcome tree shown in figure S1. These conditional probabilities may be age- and/or

sex-dependent, which implies that the age $\tilde{a}$ at disease onset of health outcome l depends on the age at infection a. The age at disease onset in turn determines disease severity and lethality and potential future health outcomes.

Let l_r be the root health outcome of ***symptomatic*** infections and $\mu(D_r \mid l_r)$ be the conditional probability of dying from health outcome l_r. Furthermore, let l_r be the root health outcome with for example maximum two[1] sequences of events (i.e. sequelae) occurring. Then $p(l_{c_i} \mid l_r)$ is the conditional probability of moving from the root health outcome l_r to the outcome l_{c_i}. There are k possible health outcomes l_{c_i} (i=1,…,k). Assume further that some of the health outcomes l_{c_i} can be followed by a health outcome $l_{g_{j(i)}}$ with m_i possible outcomes (j=1,…,m_i). Then $p(l_{g_{j(i)}} \mid l_{c_i})$ is the conditional probability of moving from health outcome c_i to health outcome $g_{j(i)}$. And $\mu(D_{c_i} \mid l_{c_i})$ is the conditional probability of dying from health outcome l_{c_i}. Assume further that $\mu(D_{g_{j(i)}} \mid l_{g_{j(i)}})$ is the conditional probability of dying from health outcome $l_{g_{j(i)}}$. Note that in most cases, many of these conditional probabilities will be 0.

For some infectious diseases also asymptomatically infected persons may develop sequelae that contribute to disease burden. While these sequelae are the same as for symptomatic infections, the conditional probability of moving from an ***asymptomatic*** infection (l_y) to health outcome l_{c_i} may be different, why denoted as $p(l_{c_i} \mid l_y)$.

If becoming infected at age a and being of sex s, than the number of non-fatal incident cases for health outcome l_{c_i} is:

$$n_{l_{c_i}}^{a,s} = n_e^{a,s} * \mathrm{p}\left(l_{c_i} \mid l_r\right)^{\tilde{a},s} + n_f^{a,s} * \mathrm{p}\left(l_{c_i} \mid l_y\right)^{\tilde{a},s} ;$$

[1] Two sequences of events are used for illustration, but *x* sequences of events can follow on symptomatic infections.

and the number of non-fatal incident cases for health outcome $l_{g_{j(i)}}$ is:

$$n_{l_{g_{j(i)}}}^{a,s} = n_{l_{c_i}}^{a,s} * \mathrm{p}(l_{g_{j(i)}} \mid l_{c_i})^{\tilde{a},s}.$$

Whereas the number of fatal incident cases for health outcome l_r is:

$$d_{l_r}^{a,s} = n_e^{a,s} * \mathrm{p}(D_r \mid l_r)^{\tilde{a},s};$$

than the number of fatal incident cases for health outcome l_{c_i} is:

$$d_{l_{c_i}}^{a,s} = n_{l_{c_i}}^{a,s} * \mathrm{p}(D_{c_i} \mid l_{c_i})^{\tilde{a},s};$$

and the number of fatal incident cases for health outcome $l_{g_{j(i)}}$ is:

$$d_{l_{g_{j(i)}}}^{a,s} = n_{l_{gj(i)}}^{a,s} * \mathrm{p}(D_{g_{j(i)}} \mid l_{g_{j(i)}})^{\tilde{a},s}.$$

Now we can combine estimated number of cases in the different health outcomes of the outcome tree with disability weight to compute disability-adjusted life years (DALYs). *YLD* for a specific health outcome *l* is calculated as the product of the duration of the illness *t* and the disability weight *w*. The YLDs for all health outcomes are then added up to get the total YLD:

$$YLD = \sum_{s=0}^{1}\sum_{a=0}^{a_{\max}}\left(\left\{n_e^{a,s} * (t_{l_r}^{\tilde{a},s} * w_{l_r}^{\tilde{a},s})\right\} + \sum_{i=1}^{k}\left(\left\{n_{l_{c_i}}^{a,s} * (t_{l_{c_i}}^{\tilde{a},s} * w_{l_{c_i}}^{\tilde{a},s})\right\} + \sum_{j=1}^{m}\left\{n_{l_{g_{j(i)}}}^{a,s} * \left(t_{l_{g_{j(i)}}}^{\tilde{a},s} * w_{l_{g_{j(i)}}}^{\tilde{a},s}\right)\right\}\right)\right)$$

Similarly, YLL due to a specific disease is calculated by summation of the number of all fatal cases (*d*) due to the health outcome (*l*) of that disease, each case multiplied by the remaining individual life expectancy (*e*) as at the age of death *ã*. Thus:

$$YLL = \sum_{s=0}^{1}\sum_{a=0}^{a_{\max}}\left(d_{l_r}^{a,s} * e_{l_r}^{\tilde{a},s}\sum_{i=1}^{k}\left(\left\{d_{l_{c_i}}^{a,s} * e_{l_{c_i}}^{\tilde{a},s}\right\} + \sum_{j=1}^{l}\left\{d_{l_{g_{j(i)^r}}}^{a_o,s} * e_{l_{g_{j(i)}}}^{\tilde{a},s}\right\}\right)\right).$$

Remaining life expectancy (*e*) as at the age of death $\tilde{a}$ is equal to the remaining life expectancy as at the age of infection *a*, but corrected for the time span in years between infection and death.

$t_l^{\tilde{a},s}$ and $e_l^{\tilde{a},s}$ have time as a unit, all other quantities are dimensionless numbers.

By adding Y*LL* and *YLD* we obtain DALYs for an infectious pathogen, including both acute illness and associated sequelae. Thus:

$$\text{DALY} = \sum_{s=0}^{1}\sum_{a_i=0}^{a_{\max}}\left(\left\{n_e^{a,s}*(t_{l_r}^{\tilde{a},s}*w_{l_r}^{\tilde{a},s})\right\}+\sum_{i=1}^{k}\left(\left\{n_{l_{c_i}}^{a,s}*(t_{l_{c_i}}^{\tilde{a},s}*w_{l_{c_i}}^{\tilde{a},s})\right\}+\sum_{j=1}^{m}\left\{n_{l_{g_{j(i)}}}^{a,s}*\left(t_{l_{g_{j(i)}}}^{\tilde{a},s}*w_{l_{g_{j(i)}}}^{\tilde{a},s}\right)\right\}\right)\right)$$

$$+\sum_{s=0}^{1}\sum_{a=0}^{a_{\max}}\left(d_{l_r}^{a,s}*e_{l_r}^{\tilde{a},s}\sum_{i=1}^{k}\left(\left\{d_{l_{c_i}}^{a,s}*e_{l_{c_i}}^{\tilde{a},s}\right\}+\sum_{j=1}^{l}\left\{d_{l_{g_{j(i)r}}}^{a_o,s}*e_{l_{g_{j(i)}}}^{\tilde{a},s}\right\}\right)\right).$$

All of the above parameters are age- and sex-dependent. Age at infection is of importance at the start of the outcome tree (i.e. root health outcome). For all later stages, it is the age of disease onset which determines the severity and the duration of the health outcome itself; and the (conditional) probability to die, to recover or to develop a sequela. There may be a functional relationship between the age at infection *a* and the age of onset of disease $\tilde{a}$, for example if disease progression is faster if infection is acquired at an older age.

For calculating numerical estimates we used point estimates for *t* and *w*. The (conditional) probabilities *p* and μ, as well as the factor(s) σ (if corrected in one step), or α and β (if corrected in two steps), as well as factor τ were sampled from uniform or pert-distributions.

Figure S1 – Outcome tree for an infectious pathogen – an illustration

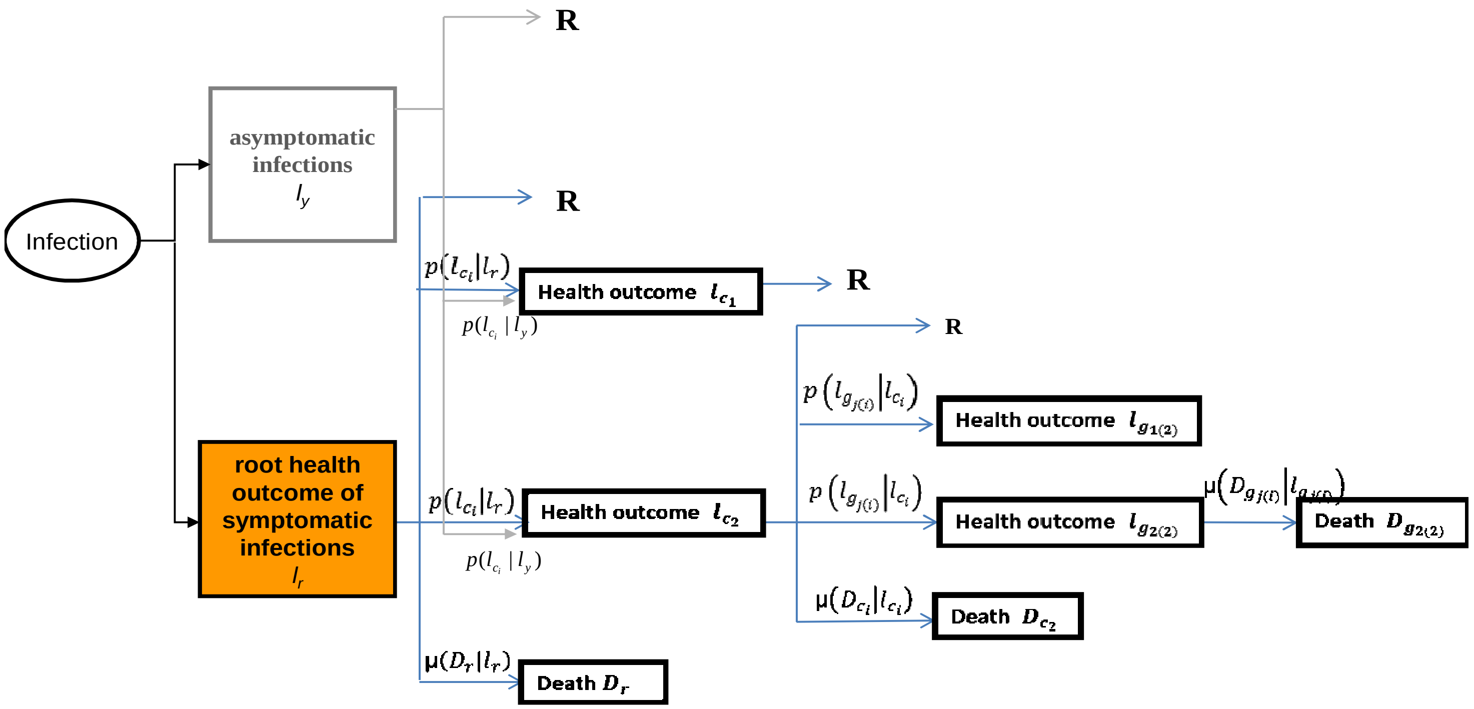

Note:

- All the above parameters are age- and sex-dependent. Age at infection is of importance at the start of the outcome tree (i.e. root health outcome). For all later stages, it is the age of disease onset which determines the severity and the duration of the health outcome; and the (conditional) probability to die, to recover or to develop a sequela.
- Two sequences of events following a symptomatic infection are used for illustration. But any number at all of sequences of events may be modelled in the here presented way.
