# Supplementary material for: The Pathogen- and Incidence-Based DALY Approach: An Appropriated Methodology for Estimating the Burden of Infectious Diseases
Source: PLoS One. 2013 Nov 20;8(11):e79740. doi: 10.1371/journal.pone.0079740 (PMC3835936; doi:10.1371/journal.pone.0079740)
Supplement: File S2 — Figures Figure S2.1 Outcome tree for Salmonella spp. Figure S2.2 Outcome tree for Campylobacter spp. Figure S2.3 Assumed age-distribution of fatal GE cases (in percentage). Figure S2.4 Assumed age-distribution of GBS cases (in percentage), as derived from Havelaar et al. Figure S2.5 The average disease burden (DALY) of Salmonella spp. and associated sequelae in the Netherlands per age-group and gender. The 95% uncertainty range is shown using error bars. Figure S2.6 The average disease burden (DALY) of Campylobacter spp. in the Netherlands per age-group and gender. The 95% uncertainty range is shown using error bars. Figure S2.7 - The distribution of undiscounted average burden of Salmonella-associated sequeleae and Campylobacter-associated sequeale, respectively, over the associated sequelae. Tables Table S2.1 - Reported laboratory-confirmed cases for Campylobacter spp. and Salmonella spp. in the Netherlands (average of the years 2005–2007). Table S2.2 -Pathogen-specific multiplication factors for the Netherlands. Table S2.3 Percentages used in the Salmonella spp. outcome tree Table S2.4 Percentages used in the Campylobacter spp. outcome tree. Table S2.5 Disability weights and duration. Table S2.6: The disease burden of Salmonella spp and associated sequelae in the Netherlands – Summary results, average and 95% CI in brackets and italic. Table S2.7: The disease burden of Campylobacter spp. and associated sequelae in the Netherlands – Summary results, average and 95% CI in brackets and italic. (PDF) [file pone.0079740.s002.pdf]

Supporting Information file 2. – Disease burden of *Salmonella* spp. and *Campylobacter* spp. in the Netherlands using the pathogen- and incidence-based DALY approach

I. Model input summary

In the following tables and figures we have summarized the model input parameters for estimating the disease burden due to *Salmonella* spp. and associated sequelae, and due to *Campylobacter* spp. and associated sequelae in the Netherlands (average 2005-2007). Full details are available on request from the first author.

Table S2.1 - Reported laboratory-confirmed cases for *Campylobacter* spp. and *Salmonella* spp. in the Netherlands (average of the years 2005-2007)

Pathogen	Reported laboratory-confirmed cases	
	Male	Female
Salmonella spp.	698.7[a,b]	728.7 [a,b]
Campylobacter spp.	1,855.7[a.b]	1,670.7 [a,b]
Source:	Laboratory surveillance [1] (personal communication by Wilfrid van Pelt; National Institute of Public Health and the Environment; June 2011)	

a) The laboratory surveillance network is composed of public laboratories that do cover in the case of *Salmonella* spp. about 64% of the Dutch population, and for *Campylobacter* spp. coverage is 52%. Correction is included in the multiplication factors.

b) Cases are stratified by age, into the age-classes: 0; 1-4; 5-9; 10-14; 15-19; 20-24; 25-29; 30-34; 35-39; 40-44; 45-49; 50-54; 55-59; 60-64; 65-69; 70-74; 75-80; 80-84 and ≥ 85 years.

Table S2.2 -Pathogen-specific multiplication factors for the Netherlands

Pathogen	Multiplication factors (MF) [a, b] *Most likely (range)*	Source
Salmonella spp.	19.8 (4.4 - 64.8)	[2]
Campylobacter spp.	18.85 (7.4 – 47.4)	[2]

a) MFs correct for underestimation (under-ascertainment and under-estimation). Additionally, the current MFs also take into account that the coverage of the lab-confirmed cases is less than 100% (see Table S2.1).

b) MFs should by definition be pathogen-, country-, age- and eventually also gender-specific (see Supporting Information file 1). But published MFs in the literature fitting Dutch laboratory-confirmed Campylobacter and Salmonella infections were only pathogen- and country-specific (for details see Gibbons et al. [3]). In the current illustration, we therefore applied for each pathogen separately the same MF to all age- and sex-classes.

Figure S2.1 Outcome tree for *Salmonella* spp.

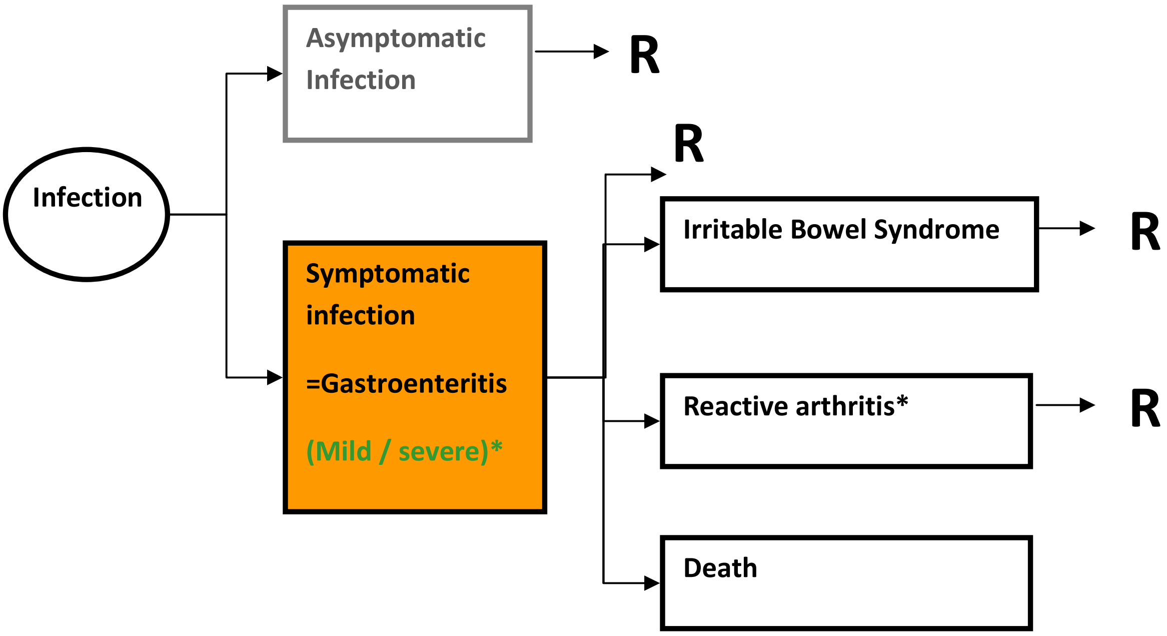

***Given the systematic uncertainty, we assumed that only “severe” Salmonella gastroenteritis (GE) cases are at risk to develop Reactive arthritis (ReA). This assumption was modified in a scenario analysis where we assumed that all Salmonella GE cases are at risk to develop ReA.

Legend used in the outcome tree:

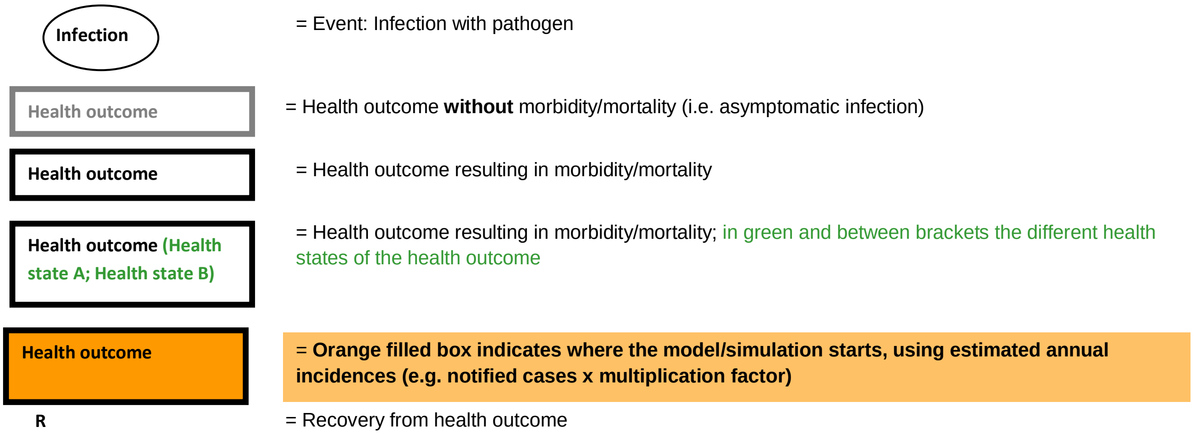

Table S2.3 Percentages used in the *Salmonella* spp. outcome tree [a]

Health outcome	Distribution of health states in health outcome *Most likely (range)*	Risk to develop that health outcome *Most likely (range)*	Sources/ Assumption
Gastroenteritis (GE)	6.24% (5.35% -7.61%) are severe GE cases	-	Laboratory-confirmed cases are a proxy for severe cases. Calculated from simulated incident cases and laboratory-confirmed salmonellosis cases in the Netherlands.
Death		0.1% (0.05%-0.3%)[b]	[4-6]
Irritable Bowel Syndrome (IBS)		8.8% (7.2% - 10.4%)	[7]
Reactive arthritis (ReA)		8% (2.3% - 15.0%)[c]	[5, 8]

a) See Figure S2.1 for the *Salmonella* spp. outcome tree.
b) See Figure S2.3 for the distribution over age-classes
c) Only severe GE cases are at risk to develop ReA.

Figure S2.2 Outcome tree for *Campylobacter* spp.

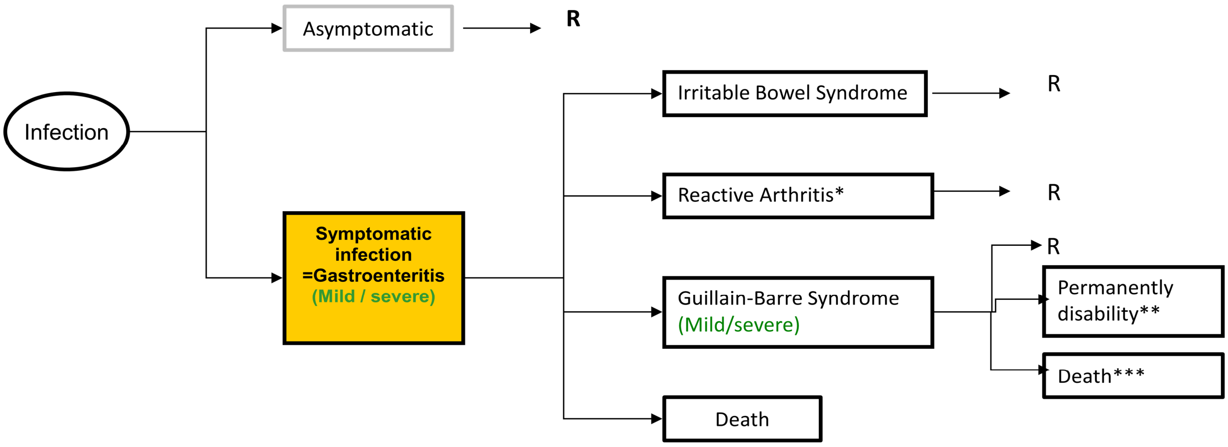

* Only severe gastroenteritis (GE) cases are at risk to develop reactive arthritis (ReA). This assumption was modified in a scenario analysis where we assumed that all Campylobacter GE cases are at risk to develop ReA. ** Non-fatal severe Guillain-Barré Syndrome (GBS) cases may develop permanent disability; *** The majority of fatal GBS cases are severe GBS cases, for reasons of simplicity we therefore assumed that only severe GBS cases may be fatal.

Legend used in the outcome tree:

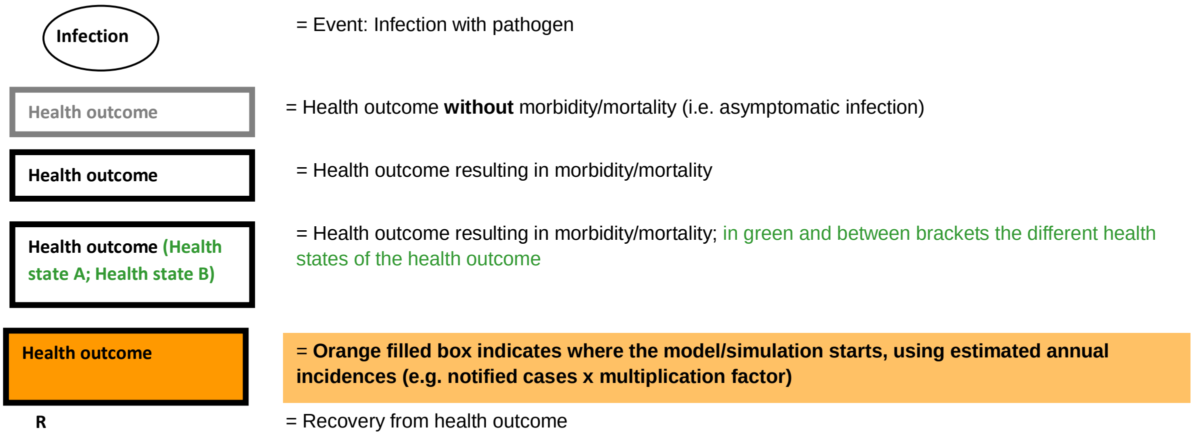

Table S2.4 Percentages used in the *Campylobacter* spp. outcome tree [a]

Health outcome	Distribution of health states in health outcome *Most likely (range)*	Risk to develop that health outcome *Most likely (range)*	Sources/ Assumption
Gastroenteritis (GE)	9.6% (4.0% - 22.6%) are severe GE cases	-	Laboratory-confirmed cases are a proxy for severe cases. Calculated from simulated number of incident cases [9] and laboratory-confirmed campylobacterosis cases in the Netherlands.
Death		0.03% (0.001%- 0.05%)[b]	[4, 9]
Irritable Bowel Syndrome (IBS)		8.8% (7.2% - 10.4%)	[7]
Reactive arthritis (ReA)		7.9% (2.4% - 8.0%)[c]	[10-12]
Guillain-Barré Syndrome (GBS)	17% are mild GBS cases[d]; 83% are severe GBS cases[d]	0.02%-0.09%[d]	[13-16]
GBS fatal		4.1% (2.4%-6.0%)[d,e]	[15-18]
GBS permanent disability		100% of non-fatal severe GBS cases	[15, 16]

a) See Figure S2.2 for the *Campylobacter* spp. outcome tree.
b) See Figure S2.3 for the distribution over age-classes
c) Only severe GE cases are at risk to develop ReA.
d) See Figure S2.4 for the distribution over age-classes
e) In about 3.4 % (2% -5%) of all GBS cases is the disease fatal [15, 18]. The majority of fatal GBS cases are hereby severe GBS cases. For simplification reasons we therefore assumed that death due to GBS occurs only in severe cases, resulting in 4.1% (2.4% - 6.0%).

Table S2.5 Disability weights and duration

Health outcome (health state)	Disability weight (w)		Duration	
	w	Source	in years	Source
GE (health outcome)	0.105	[19]	0.017	[5]
IBS	0.042	[20]	5	[7]
ReA	0.059	Calculated based on Haagsma *et al.* [20] and Hannu *et al.* [10]	1 [a]	[20]
GBS (mild cases); clinical phase	0.090	[21]	1 [a]	[21]
GBS (severe cases); clinical phase	0.280	[21]	1 [a]	[21]
GBS (severe cases) permanent disability	0.160	[21]	Remaining Life Expectancy	[21]

a) Duration based on an annual profile disability weight

Figure S2.3 Assumed age-distribution of fatal GE cases (in percentage)

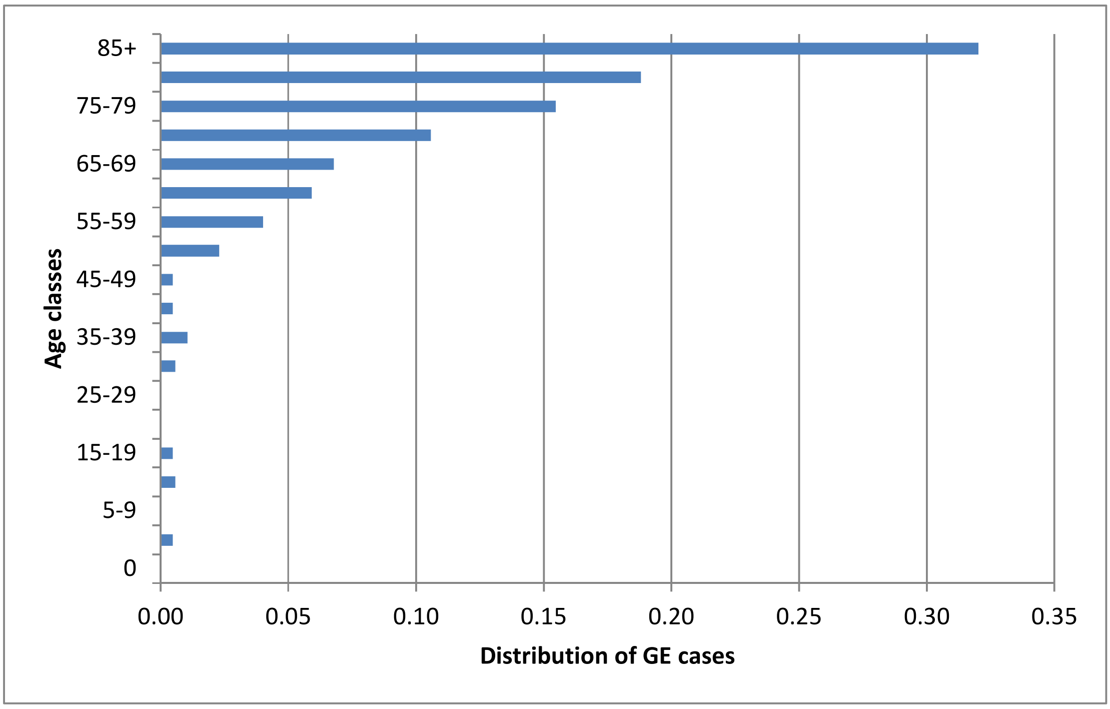

Source: Based on all reported fatal Salmonellosis cases in Estonia, Germany and the Netherlands for the years 2005-2007

Figure S2.4 Assumed age-distribution of GBS cases (in percentage), as derived from [15,16].

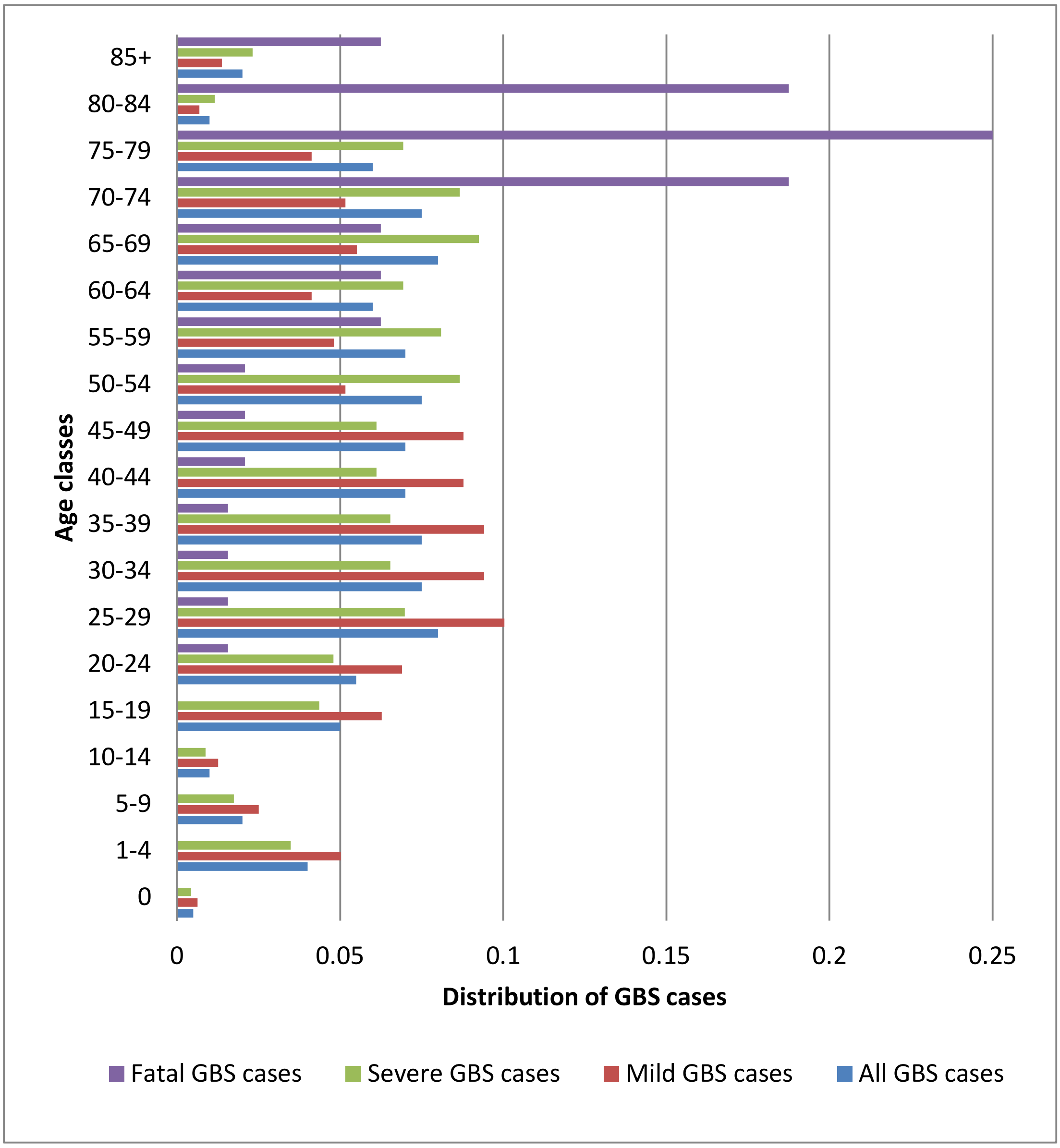

Note: Fatal GBS cases are mostly severe GBS cases. For simplification reason we therefore assumed that only severe GBS cases are fatal.

II. Results

In the following tables and figures we have summarized some of the estimated results for *Salmonella* spp. and associated sequelae, and for *Campylobacter* spp and associated sequelae in the Netherlands (average 2005-2007). The results show undiscounted DALYs with uniform age-weights.

Table S2.6: The disease burden of *Salmonella* spp and associated sequelae in the Netherlands – Summary results, average and 95% CI in brackets and italic.

Health outcome(s)	Cases	YLD per year	YLL per year	DALY per year	DALY per case *Symptomatic*	DALY per 100,000
**ACUTE ILLNESS**						
**GE (sum)**		**66**	**462**	**529**	**0.015**	**3.2**
		(55-78)	*(261-728)*	*(325-798)*	*(0.010-0.022)*	*(1.99-4.89)*
Cases	35,300	66		**66**		
	(29,250-41,680)	*(55-78)*		*(55-78)*		
Death	44		462	**462**		
	(25-70)		*(261-728)*	*(261-728)*		
**SEQUELAE**						
**IBS (sum)**		**652**	**0**	**652**	**0.018**	**4.0**
		(529-790)		*(529-790)*	*(0.017-0.020)*	*(3.2-4.8)*
Cases	3,110	652		**652**		
	(2,520-3,760)	*(529-790)*		*(529-790)*		
**ReA (sum)**		**11**		**11**	**0.0003**	**0.07**
		(6-16)	**0**	*(6-16)*	*(0.0002-0.0004)*	*(0.04-0.09)*
Cases	183	11		**11**		
	(107-273)	*(6-16)*		*(6-16)*		
**Sequelae (sum)**		**663**	**0**	**663**	**0.019**	**4.1**
		(538-803)		*(538-803)*	*(0.017-0.021)*	*(3.3-4.9)*
**ALL health outcomes**		**730**	**462**	**1,192**	**0.034**	**7.3**
		(594-879)	*(261-728)*	*(913-1,530)*	*(0.028-0.041)*	*(5.6-9.4)*

Note: Total infected cases are not reported as asymptomatic salmonella cases were not explicitely modelled. Reason for not considering asymptomatic infections was that they do not contribute to the disease burden.

Figure S2.5 The average disease burden (DALY) of *Salmonella* spp. and associated sequelae in the Netherlands per age-group and gender. The 95% uncertainty range is shown using error bars.

Male	Female
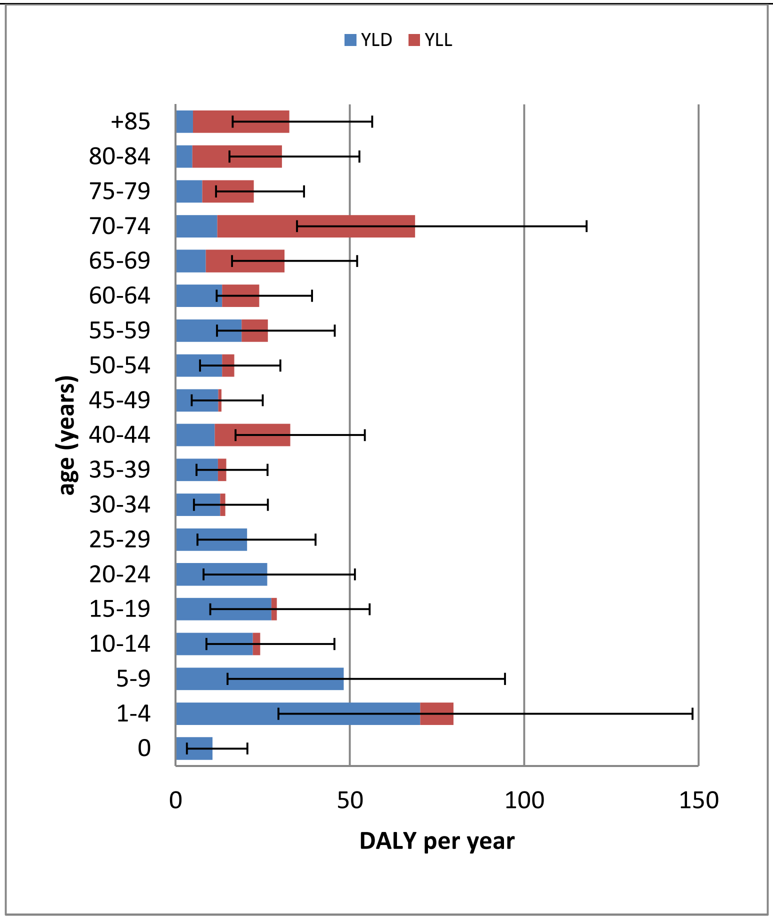	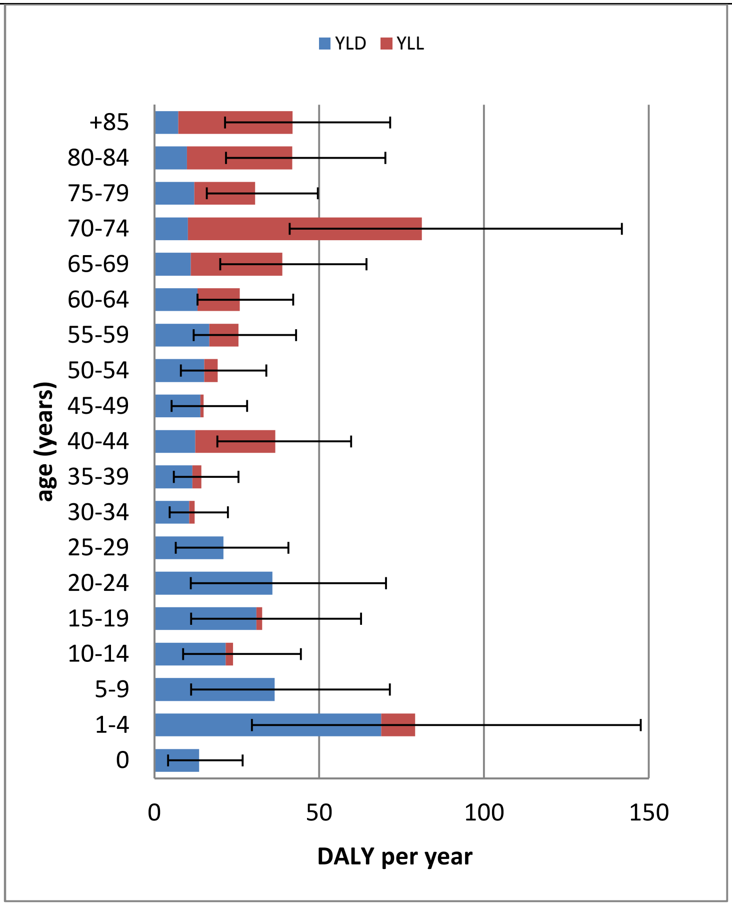

Table S2.7: The disease burden of *Campylobacter* spp. and associated sequelae in the Netherlands – Summary results, average and 95% CI in brackets and italic

Health outcome(s)	Cases	YLD per year	YLL per year	DALY per year	DALY per case *Symptomatic*	DALY per 100,000
**ACUTE ILLNESS**						
**GE (sum)**		**144**	**227**	**371**	**0.005**	**2.3**
		(127-160)	*(122-328)*	*(262-479)*	*(0.003-0.006)*	*(1.6-2.9)*
Cases	76,500	144		**144**		
	(67,800-85,550)	*(127-160)*		*(127-160)*		
Death	22		227	**227**		
	(12-31)		*(122-328)*	*(122-328)*		
**SEQUELAE**						
**IBS (sum)**		**1,414**	**0**	**1,414**	**0.018**	**8,7**
		(1,214-1,635)		*(1,214-1,635)*	*(0.017-0.020)*	*(7.4-10.0)*
Cases	6,700	1,414		**1,414**		

	(5,800-7,800)	*(1,214-1,635)*		*(1,214-1,635)*		
**ReA (sum)**		**34**	**0**	**34**	**0.0004**	**0.2**
		(20-52)		*(20-52)*	*(0.0003-0.0007)*	*(0.1-0.3)*
Cases	581	34		**34**		
	(335-889)	*(20-52)*		*(20-52)*		
**GBS (sum)**		**191**	**50**	**242**	**0.003**	**1.5**
		(76-314)	*(18-91)*	*(94-398)*	*(0.001-0.005)*	*(0.6-2.4)*
Cases	42	191		**191**		
	(18-67)	*(76-314)*		*(76-314)*		
Death	1.4		50	**50**		
	(0.5-2.6)		*(18-91)*	*(18-91)*		
**Sequelae (sum)**		**1,640**	**50**	**1,690**	**0.022**	**10.3**
		(1,379-1,920)	*(18-91)*	*(1,406-1,994)*	*(0.019-0.025)*	*(8.6-12.2)*
		**1,783**	**277**	**2,061**	**0.027**	**12.6**
**ALL Health Outcomes**		*(1,512-2,075)*	*(165-387)*	*(1,737-2,406)*	*(0.024-0.030)*	*(10.6-14.7)*

Note: Total infected cases are not reported as asymptomatic campylobactera cases were not explicitely modelled. Reason for not considering asymptomatic infections was that they do not contribute to the disease burden.

Figure S2.6 The average disease burden (DALY) of *Campylobacter* spp. in the Netherlands per age-group and gender. The 95% uncertainty range is shown using error bars.

Male	Female
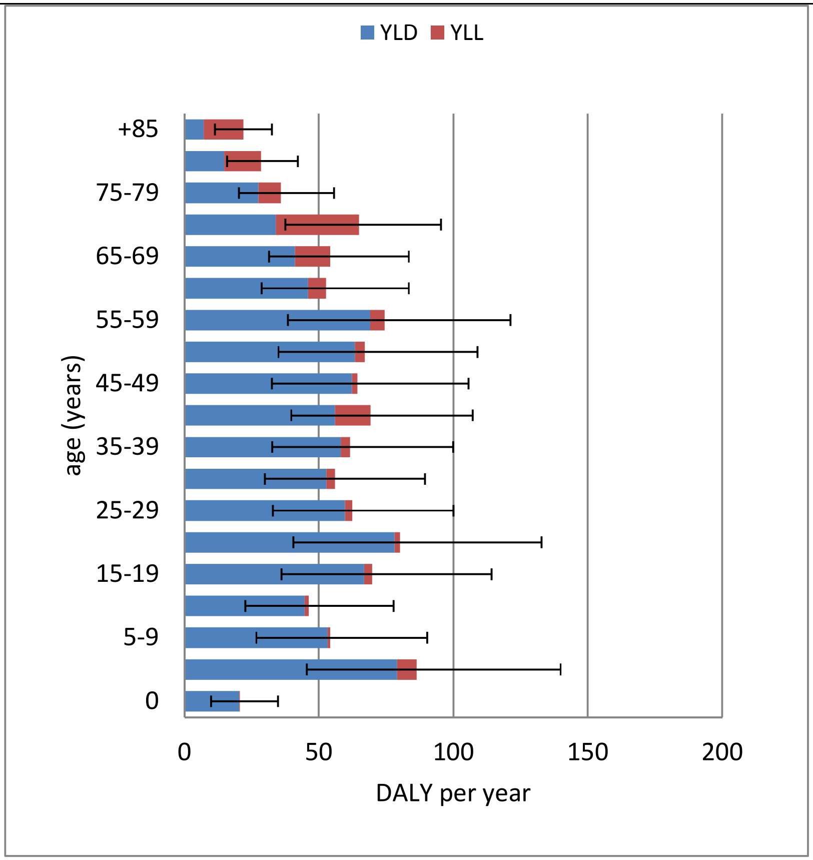	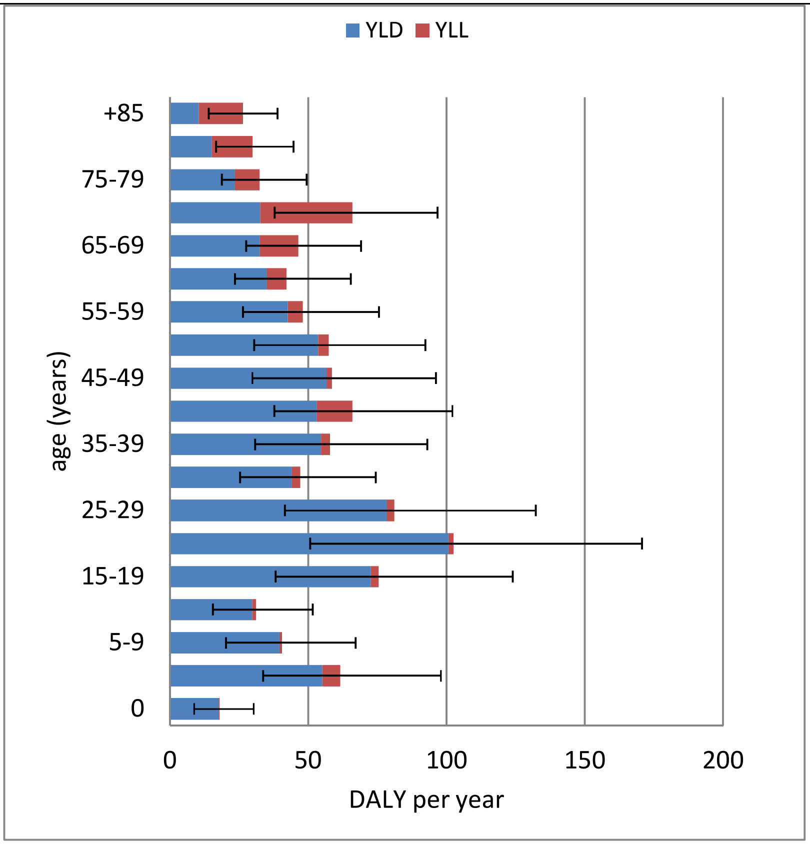

Figure S2.7 - The distribution of undiscounted average burden of Salmonella-associated sequeleae and Campylobacter-associated sequeale, respectively, over the associated sequelae.

Salmonella-associated sequelae	Campylobacter-associated sequelae
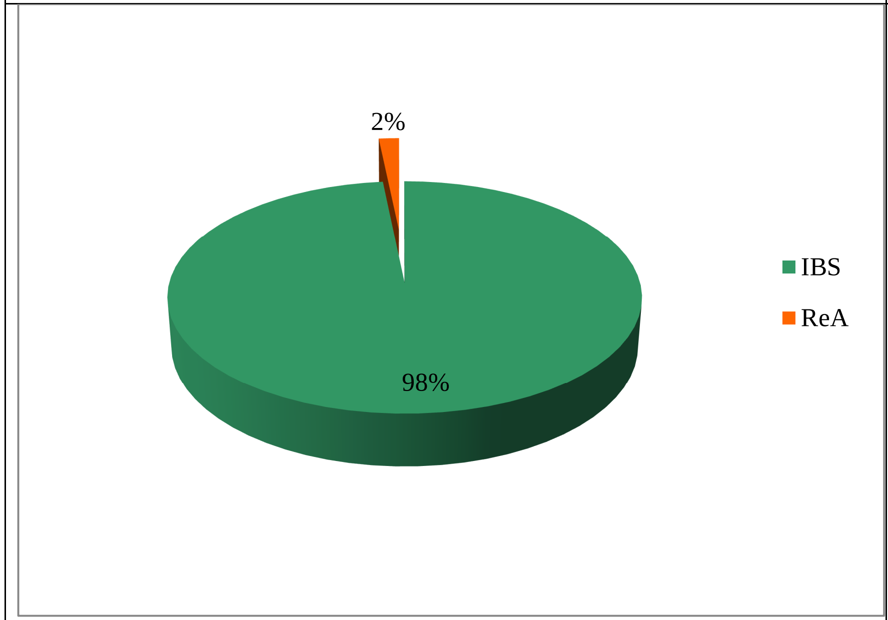	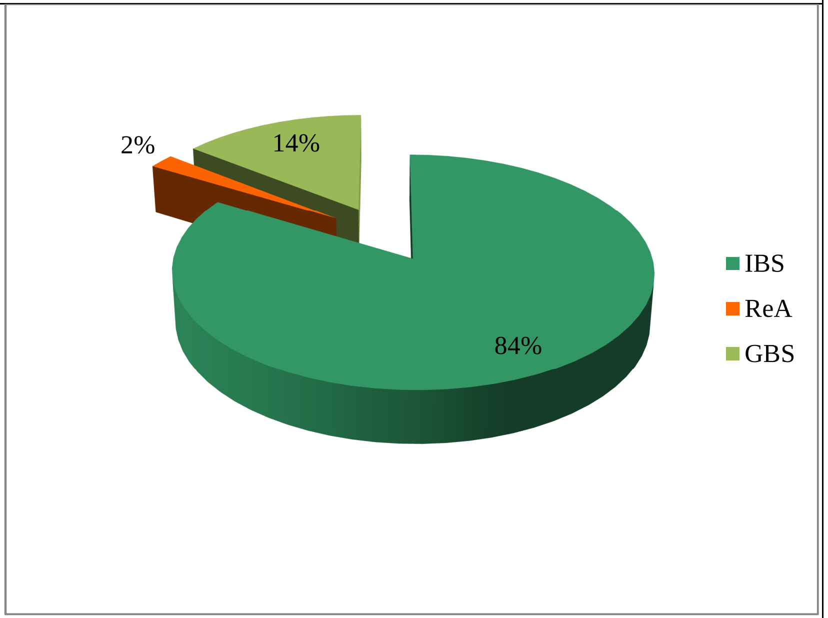

References

[1] van Pelt W, de Wit MA, Wannet WJ, Ligtvoet EJ, Widdowson MA, van Duynhoven YT. Laboratory surveillance of bacterial gastroenteric pathogens in The Netherlands, 1991-2001. Epidemiol Infect 2003; 130(3):431-41.

[2] Havelaar AH, Ivarsson S, Löfdahl M, Nauta MJ. Estimating the true incidence of campylobacteriosis and salmonellosis in the EU. Epidemiol Infect 2013; 141 (2): 293-302.

[3] Gibbons CL, Mangen MJJ, Plass D, Havelaar AH, Brooke RJ, Cassini A, Peterson K, Stuurman A, Fèvre E, Kretzschmar MEE. Estimating the extent of under-reporting and under-ascertainment of infectious diseases. (In preparation).

[4] Adak GK, Long SM, O'Brien SJ. Trends in indigenous foodborne disease and deaths, England and Wales: 1992 to 2000. Gut 2002; 51(6):832-41.

[5] Kemmeren JM, Mangen M-JJ, van Duynhoven YTHP, Havelaar AH. Priority setting of foodborne pathogens: disease burden and costs of selected enteric pathogens. Bilthoven: National Institute of Public Health and Environment; 2006.

[6] Much P, Pichler J, Allerberg F. Food borne infectious outbreaks, Austria 2005. Wien Klin Wochenschr 2007; 119(5-6):150-7.

[7] Haagsma JA, Siersema PD, De Wit NJ, Havelaar AH. Disease burden of post-infectious irritable bowel syndrome in the Netherlands. Epidemiol Infect 2010; 138(11):1650-6.

[8] Raybourne RB, Williams KM, Roberts T, and Arthritis Working Group. Food Poisoning: Economic Implications. In: Caballero B, Trugo L, Finglas P (eds). Encylopedia of Food Sciences and Nutrition. London; 2003.

[9] Mangen MJJ, Havelaar AH, Bernsen RAJAM, Van Koningsveld R, de Wit GA. The costs of human Campylobacter infections and sequelae in the Netherlands: a DALY and cost-of-illness approach. Food economics - Acta Agriculturae Scandinavica, Section C 2005; 2(1):35-51.

[10] Hannu T, Mattila L, Rautelin H, Pelkonen P, Lahdenne P, Siitonen A, et al. Campylobacter-triggered reactive arthritis: a population-based study. Rheumatology 2002; 41(3):312-8.

[11] Kosunen TU, Kauranen O, Martio J, Pitkanen T, Ponka A, Hortling L, et al. Reactive arthritis after campylobacter jejuni enteritis in patients with HLA-B27. Lancet 1980; 1(8181):1312-3.

[12] Johnsen K, Ostensen M, Melbeye AC, Melby K. HLA-B27-negative arthritis related to Campylobacter jejuni enteritis in three children and two adults. Acta Med Scand 1983; 214(2):165-8.

[13] Allos BM. Association between Campylobacter infection and Guillain-Barré syndrome. J Infect Dis 1997; 176(Suppl2):S125-S8.

[14] Ternhag A, Törner A, Svensson A, Ekdahl K, Giesecke J. Short- and long-term effects of bacterial gastrointestinal infections. Emerg Infect Dis 2008; 14(1):143-8.

[15] Havelaar AH, de Wit MAS, van Koningsveld R, van Kempen E. Health burden in the Netherlands due to infection with thermophilic Campylobacter spp. Epidemiol Infect 2000; 125(3):505-22.

[16] Havelaar AH, de Wit MAS, van Koningsveld R. Health burden in the Netherlands (1990-1995) due to infection with thermophilic Campylobacter species. Bilthoven: National Institute for Public Health and the Environment; 2000.

[17] Van Koningsveld R, Van Doorn PA, Schmitz PI, Ang CW, Van der Meche FG. Mild forms of Guillain-Barre syndrome in an epidemiologic survey in The Netherlands. Neurology 2000; 54(3):620-5.

[18] Van Koningsveld R, Van Doorn PA, Schmitz PI, Van der Meche FG. Changes in referral pattern and its effect on outcome in patients with Guillain-Barre syndrome. Neurology 2001; 56(4):564-6.

[19] WHO. Global Burden of Disease 2004 Update: Disability weights for diseases and conditions. Geneva: WHO; 2004.

[20] Haagsma JA, Havelaar AH, Janssen BM, Bonsel GJ. Disability Adjusted Life Years and minimal disease: application of a preference-based relevance criterion to rank enteric pathogens. Popul Health Metr 2008; 6:7.

[21] Havelaar AH, Haagsma J, Mangen MJJ, Kemmeren JM, Verhoef L, Vijgen SMC, et al. Disease burden of foodborne pathogens in the Netherlands, 2009. Int. J Food Microbiol. 2012; 156(3):231-8.
